# A systematic and meta-analysis of heterosexual behaviors and HIV prevalence among Chinese men who have sex with men

**DOI:** 10.1186/s12981-021-00392-6

**Published:** 2021-10-10

**Authors:** Huan Zhao, Xi Li, Junhua Wang, Wen Wang, Chunyan Yang, Jingjuan Li, Xiuling Li, Rui Pu, Lulu Chen, Xiao Zhang, Jun Zhang, Peng Luo, Jiangping Zhang

**Affiliations:** 1 School of Public Health, Key Laboratory of Environmental Pollution Monitoring Control ministry of Education, Guizhou Medical University, Guiyang, 550000 People’s Republic of China; 2 Center for Disease Control and Prevention of Yunyan of Guiyang City, Guizhou, People’s Republic of China; 3 State Key Laboratory of Functions and Applications of Medicinal Plants, Guizhou Medical University, Guiyang, 550014 People’s Republic of China; 4 Yunyan District Health Bureau of Guiyang City, D12 in Weilaifangzhou, Yunyan District, Guiyang, 550000 Guizhou People’s Republic of China; 5 School of Public Health, Key Laboratory of Environmental Pollution Monitoring Control Ministry of Education, Guizhou Medical University, Town of University, Guian New District, Guiyang, 550025 People’s Republic of China

**Keywords:** Men who have sex with men, Heterosexual behavior, Condom use, HIV prevalence, Meta-analysis

## Abstract

Men who have sex with men (MSM) are potentially at high risk for HIV infection, their HIV prevalence far exceeds the national prevalence rate. There are also a number of MSM who have sex with women, mostly unprotected, which can transmit HIV to their female sexual partners and even to the next generation. The purpose of this article is to evaluate the prevalence of heterosexual behaviors in Chinese men who have sex with men and the status of condom utilization as well as HIV infection among MSM with heterosexual behaviors, so as to further improve the investigation and prevention and control of AIDS in MSM population. We systematically searched China National Knowledge Infrastructure, Wanfang Data, VIP Database for Chinese Technical Calligraphy (VIP), Pubmed, and Embase following certain retrieval strategies to find relevant articles published from January 1, 2015 to November 18, 2019, The useful information extracted from qualified articles, Stata 15.1 and Review Manager 5.3 were employed for further meta analysis. The estimated prevalence of heterosexual behaviors among MSM in the past year was 19.0% (95% CI 17.0%, 22.0%). The estimated condom utilization rate of the last heterosexual behavior among MSM and condom adherence rate of heterosexual behaviors among MSM were 51.0% (95% CI 44.0%, 58.0%) and 31.0% (95% CI 25.0%, 38.0%), respectively. And the pooled HIV prevalence in MSM with heterosexual behaviors was 9% (95% CI 6%, 13%). The sensitivity analysis showed that the results were stable. No publication bias was found by Egger’s test. There is a high proportion of MSM with heterosexual behaviors and low condom utilization in China. HIV prevalence in MSM with heterosexual behaviors is also high. Therefore, adequate attention should be given to this particular group and measures should be taken in order to reduce the risk of transmission of HIV from subpopulations to the general population.

## Text

### Introduction

Acquired immunodeficiency syndrome (AIDS) is an infectious disease with a high fatality rate caused by the human immunodeficiency virus (HIV). HIV destroys the body’s immune system, making infected people gradually lose their ability to fight off various diseases and eventually lead to death. There is no vaccine to prevent it and no effective drug to cure the disease. AIDS seriously endangers human health and hinders the stable development of society. “AIDS is not only a medical problem, but also a major public health problem, but also a serious social problem,“ the statement is accepted around the world. HIV infection varies markedly at national levels. Countries in southern sub-Saharan Africa had HIV prevalence rates of more than 10% in 2017. China’s overall HIV infection rate remains at a low level. It was estimated at 0.058 percent (0.046–0.070%) of the total population by the end of 2011. The total number of hiv-infected people reported to be alive accounted for 0.05% of the total population by the end of 2017. However, a high prevalence of HIV in confined areas, such as Yunnan and Guangdong, and specific groups ,such as drug users, MSM, in China [1]. It is worth noting that the positive rate of HIV antibody among MSM in China has been increasing rapidly in recent years,which was 3.0% in 2003, 5.7% in 2010, 7.3% in 2013 and 7.98% in 2015 [2]. MSM population is young and sexually active [3, 4], who usually have anal sex. Besides, anal sex is most likely to lead to the infection of HIV than vaginal sex because the anal mucosa is thin and prone to injury. MSM are characterized by multiple sexual partners and low condom utilization with their own social networks that are hard to get [5]. For these reasons, MSM forms the potentially high-risk group for HIV infection.

According to the report of Joint United Nations Programme on HIV/AIDS (UNAIDS), the key populations, including MSM and their sexual partners accounted for more than half (54%) of new HIV infections worldwide in 2018 [6]. It is reported by Several provinces in China that sexual transmission accounted for more than 90% of HIV infections and AIDS patients in 2019, and the constituent ratio of homosexual transmission varied from 6.5 to 75.66%, but this was higher than that of the previous years [7]. A certain number of MSM had or are having a sexual relation with women, which may transmit HIV to the female sexual partners and even to the next generation [8]. This study made a meta-analysis on the prevalence of heterosexual behaviors among MSM population, condom utilization and HIV prevalence in MSM population with heterosexual behaviors to understand the behavioral characteristics and HIV infection risk of MSM population with heterosexual behaviors, thereby providing a basis for targeted AIDS prevention and treatment among MSM population.

## Method

### Date sources and search strategy

Electronic databases such as CNKI, Wanfang, VIP, PubMed and Embase were searched on a systematic basis. The relevant studies published from January 1, 2015 to November 18, 2019 were retrieved from the above databases with the keywords and medical subject headings of “acquired immune deficiency syndrome”, “human immunodeficiency virus infection”, “MSM”, “bisexuality”, “bisexual men”, “MSMW”, and “men who have sex with men and women” .

### Inclusion and exclusion criteria

The included studies were based on the following criteria: (1) cross-sectional studies or baseline studies in cohort studies; (2) participants were Chinese MSM who had oral or anal sex in the past year. (3) ages ≥ 16; (4) reported the outcome indicators, i.e. the prevalence of heterosexual behaviors among MSM in the past year, the condom utilization rate of the last heterosexual behavior among MSM, condom adherence rate of heterosexual behaviors among MSM or HIV prevalence in MSM with heterosexual behaviors, the above indicators could be calculated indirectly from the data given in studies. Exclusion criterias: (1) duplicated studies or studies based on the same data source; (2) reviews, news reports, and animal experiments; (3) post-intervention studies; (4) outcome indicators not reported; (5) Heterosexual behaviors recall windows of MSM population not reported or lasted for more than 1 year.

### Study selection and data extraction

Two independent reviewers selected the articles based on the inclusion criteria, and extracted data on the first author, published year, year of study, study locations, setting, recall window of heterosexual behaviour among MSM, sampling method, survey method, sample size and reported outcome indicators with 95% confidence intervals (CIs). Moreover, we discussed any differences before reaching an agreement at last.

### Quality assessment

AHRQ cross-sectional study evaluation standards were selected for the evaluation of the quality of the included studies, which consists of eleven items, specifically, a score of 1 represents “yes” and 0 refers to “no” or “unclear”. The total score is between 0 and 3, 4 and 7, as well as 8 and 11 respectively denotes “bad”, “satisfactory” and “good ” in terms of quality.

### Statistical analysis

The prevalence of heterosexual behaviors among MSM and its 95% CIs extracted from studies were pooled by using the fixed-effect model or the random-effect model according to heterogeneity between studies. Similarly, a summary estimate was made for the condom utilization rate of last heterosexual behavior, condom adherence rate of heterosexual behaviors, and HIV prevalence in MSM with heterosexual behaviors.

The existence and degree of heterogeneity were detected by Cochran’s Q test and I^2^ statistical values. For the Q statistic, there was heterogeneity at *P* < 0.05; and in the case that the I^2^ statistic and I^2^ values are less than 25%, it indicates low heterogeneity, while the percentages of 50% and 75% indicate medium and high heterogeneity, respectively. If the results of the tests agree with the hypothesis of homogeneity, the fixed-effect model using inverse arcsine variance weights were used for the estimation of the overall prevalence across studies, otherwise random-effect model using DerSimoniane-Laird (D-L) weight would be used. The differences in prevalence reported in these studies may be due to differences in characteristics of the target population and the methodology. Therefore, an analysis on a subgroup of study locations, setting, sampling method, survey method, recall windows of heterosexual behaviors among MSM, sample size, study year was performed for the detection of potential sources of heterogeneity.

A sensitivity analysis was made by excluding one study at a time to check the stability of the outcomes and the sources of heterogeneity. The funnel plot was utilized to preliminarily judge whether there was publication bias, and further quantitative judgment was determined by Egger’s test. The statistical softwares of Stata version 15 and Review Manager version 5.3 were employed for further quantitative meta-analysis.

## Results

### Search and selection of studies

**Fig. 1:**
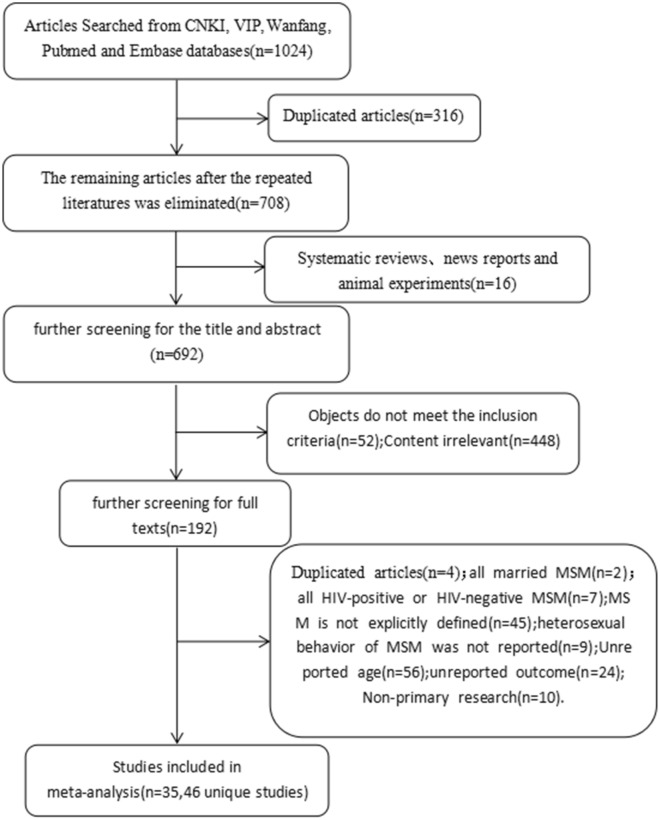
Flow chart of articles section for systematic review

A total of 1024 articles were eventually retrieved from the above databases. After the elimination of duplicates, 708 articles were retained. These articles went through two stages of screening. In the first stage, by reading the titles and abstracts and excluding articles failed to meet the inclusion criteria, 192 articles were included in the second stage, of which the full text was required to be read the full text. After excluding the articles in which, for example, the participants were all HIV-infected or negative, or the cases were without reported outcome indicators; at last, a total of 35 articles (46 independent studies) were included in this meta-analysis (Fig. 1).

There are 46 independent studies including 27,579 MSM, with the largest sample size of 2377 and the smallest one of 117. The studies covered 17 provinces or municipalities. In fact, 36.9% of the studies were conducted in the prosperous eastern China regions of Shanghai, Jiangsu, Zhejiang, Anhui, Fujian, Jiangxi and Shandong, respectively, and 23.9% of the studies were made in southern China, such as Guangdong, Guangxi and Hainan. The studies involved a variety of recruitment methods, such as working groups, MSM venues, non-governmental organizations (NGOs) and voluntary counseling and testing (VCT). Non-probability sampling was employed in these among studies, such as snowball sampling and convenience sampling. The surveys were mainly achieved by using face-to-face interviews and self-administered questionnaires, which were popular at present. For all the included studies, the descriptive characteristics are listed in Table 1, and the quality of included studies was evaluated as per through the AHRQ cross-sectional study evaluation standards with the scores shown in Table 1. The scores of all the included studies after evaluation ranged from 5 to 8 points, in another word, the quality of the included articles was of satisfactory or good.

**Table 1:** The descriptive characteristics of included studies

First author and Publication year	Study year	Study locations	Setting	Sampling method	Survey method	Recall windows(months)	Sample size	AHRQ score
Zhu, 2019-1[9]	2015	Guangdong	VCT	Not reported	Face-to-face	6	520	5
Zhu, 2019-2 [9]	2016	Guangdong	VCT	Not reported	Face-to-face	6	516	5
Zhu, 2019-3 [9]	2014	Guangdong	VCT	Not reported	Face-to-face	6	163	5
Zhu, 2019-4 [9]	2015	Guangdong	VCT	Not reported	Face-to-face	6	357	5
Zhu, 2019-5 [9]	2016	Guangdong	VCT	Not reported	Face-to-face	6	344	5
Yu, 2019-1 [10]	2017	Sichuan	Multiple recruitment methods	Not reported	Face-to-face	6	410	5
Yu, 2019−2 [10]	2017	Sichuan	Multiple recruitment methods	Snowball sampling	Face-to-face	6	460	5
Wu, 2019 [11]	Not reported	Guangdong	VCT	Snowball sampling + RDS	Face-to-face	6	434	8
Pan, 2019-1 [12]	2015	Guizhou	Multiple recruitment methods	Not reported	Face-to-face	6	248	5
Pan, 2019-2 [12]	2016	Guizhou	Multiple recruitment methods	Not reported	Face-to-face	6	250	5
Pan, 2019-3 [12]	2017	Guizhou	Multiple recruitment methods	Not reported	Face-to-face	6	252	5
Pan, 2019-4 [12]	2018	Guizhou	Multiple recruitment methods	Not reported	Face-to-face	6	253	5
Duan, 2019 [13]	2016	Shandong	Multiple recruitment methods	Snowball sampling	Face-to-face	6	1306	5
Ding, 2019 [14]	2014	Henan	MSM venues	Snowball sampling	Not reported	6	205	5
Zeng, 2019 [15]	2016	Guangxi	Social organization	Convenience sampling	Face-to-face	6	375	6
Wu, 2019 [16]	2016–2017	Hunan	Social organization	Not reported	Web based	6	556	5
Tang, 2018 [17]	2013	Zhejiang	Multiple recruitment methods	Convenience sampling	Face-to-face	3	238	5
Sun, 2018 [18]	2015–2016	Guangdong	Multiple recruitment methods	Snowball sampling	Self-administered	6	334	6
Hu, 2018 [19]	2017	Chongqing	MSM venues	Not reported	Face-to-face	12	300	6
Guo, 2018 [20]	2013–2015	Jiangsu	Multiple recruitment methods	Snowball sampling	Face-to-face	6	1895	6
Lin, 2017 [21]	2013–2015	Zhejiang	MSM venues	Convenience sampling	Face-to-face	6	1399	5
Guo, 2017 [22]	2013–2015	Jiangsu	Not reported	Snowball sampling	Face-to-face	6	2377	6
Wang, 2016 [23]	Not reported	Yunnan	NGO	Snowball sampling + RDS	Face-to-face	6	557	5
Qiu, 2016 [24]	2012–2015	Hubei	Not reported	Snowball sampling	Face-to-face	6	1559	5
Li 2016 [25]	2014–2016	Guangdong	MSM venues	TLS + Snowball sampling	Not reported	6	801	5
Lei, 2016 [26]	2014	Hunan	NGO	Snowball sampling RDS	Not reported	6	604	6
Gong, 2016 [27]	2011–2015	Shandong	MSM venues	Not reported	Face-to-face	6	1350	6
Du, 2016 [28]	Not reported	Jiangxi	Not reported	Not reported	Self-administered	6	117	5
Ding, 2016 [29]	2014–2015	Zhejiang	Working group	Snowball sampling + RDS	Face-to-face	3	351	5
Zhao, 2015 [30]	2012–2014	Liaoning	Not reported	Snowball sampling	Face-to-face	6	1208	5
Zhang, 2015 [31]	2009	Yunnan	Not reported	Snowball sampling	Face-to-face	6	453	6
Zhang, 2015 [32]	Not reported	Shanghai	Social organization	Snowball sampling	Face-to-face	6	673	5
Wang, 2015 [33]	2013	Liaoning	Not reported	RDS	Face-to-face	6	900	5
Wang, 2015 [34]	2013–2014	Shandong	Not reported	Not reported	Face-to-face	6	408	5
Tang, 2015 [35]	Not reported	Zhejiang	Multiple recruitment methods	RDS	Not reported	3	238	5
Sun, 2015 [36]	2014	Not reported	working group	non-probability sampling	Face-to-face	6	524	5
Ren, 2015 [37]	2008–2009	Shaanxi	Not reported	Snowball sampling	Not reported	6	1365	5
Peng, 2015 [38]	2013–2014	Guangdong	Not reported	Convenience sampling	Face-to-face	6	369	6
Long, 2015 [39]	2014	Guangdong	Multiple recruitment methods	Snowball sampling	Face-to-face	6	421	5
Li, 2015 [40]	Not reported	Heilongjiang	Multiple recruitment methods	Snowball sampling	Face-to-face	6	400	5
Gong, 2015-1 [41]	2010	Shandong	Multiple recruitment methods	Snowball sampling	Face-to-face	6	310	5
Gong, 2015-2 [41]	2011	Shandong	Multiple recruitment methods	Snowball sampling	Face-to-face	6	350	5
Gong, 2015-3 [41]	2012	Shandong	Multiple recruitment methods	Snowball sampling	Face-to-face	6	402	5
Gong, 2015-4 [41]	2013	Shandong	Multiple recruitment methods	Snowball sampling	Face-to-face	6	364	5
Gong, 2015-5 [41]	2014	Shandong	Multiple recruitment methods	Snowball sampling	Face-to-face	6	329	5
Chen, 2018 [42]	2010–2017	Zhejiang	Not reported	Non-probability sampling	Not reported	6	334	5

### Estimated prevalence of heterosexual behaviors among MSM

**Fig. 2:**
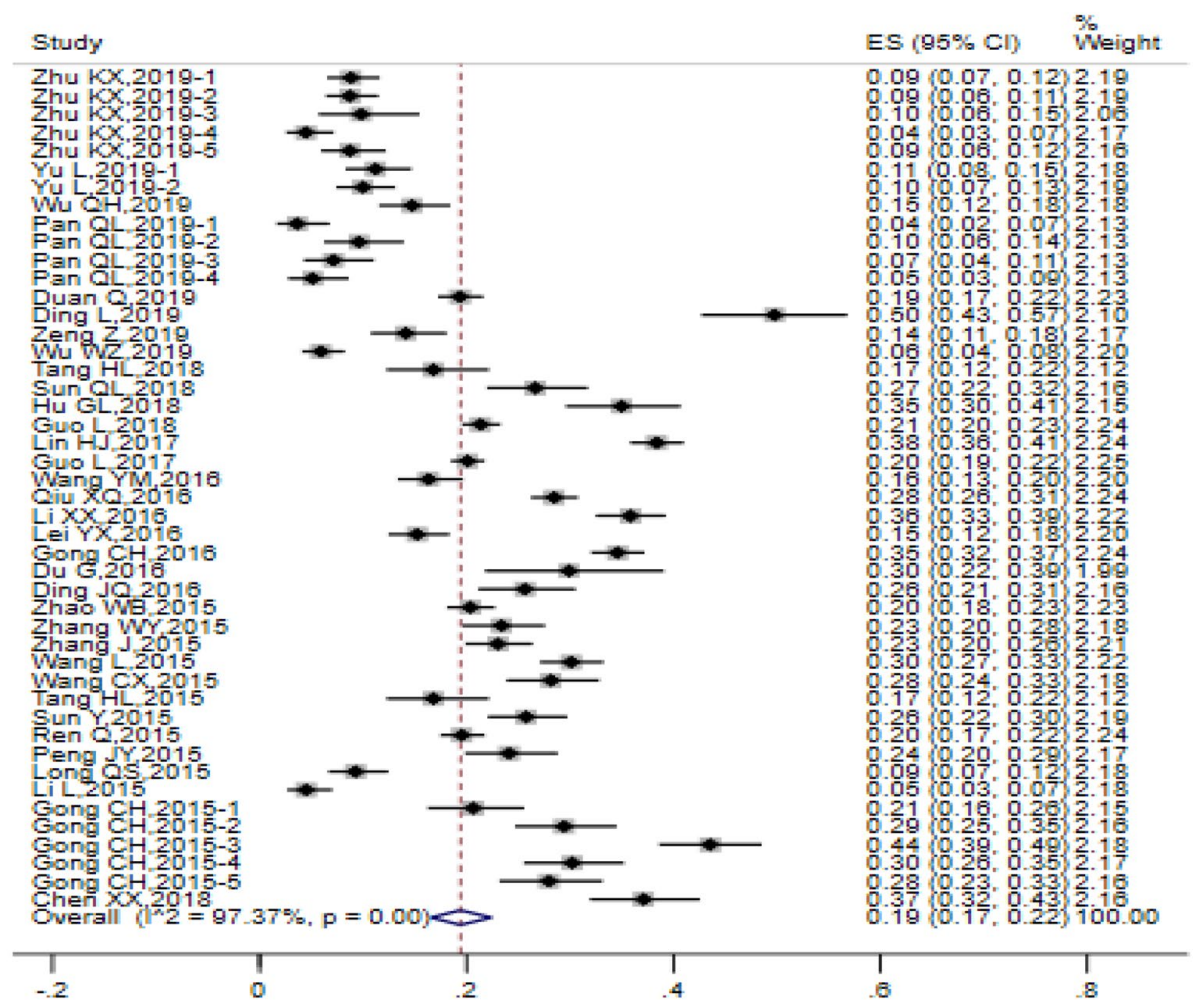
Forest chart of the prevalence of heterosexual behaviors among MSM

High heterogeneity was observed among the 46 studies (I^2^ = 97.37%, Q test *P* < 0.0001). D-L weight of random-effect model was employed for the estimation of the prevalence of heterosexual behaviors among MSM and the pooled prevalence was 0.19 (95% CI 0.17, 0.22). The forest chart is illustrated in Fig. 2.

### Subgroup analysis

To explore the sources of heterogeneity in the prevalence of heterosexual behaviors among MSM, we conducted a subgroup analysis of the study locations, setting, sampling method, survey method, recall windows of heterosexual behaviors among MSM, sample size, and study year (Table 2). In general, heterogeneity remained almost unchanged after subgroup analysis, but the relevant information was still obtainable. The prevalence of heterosexual behaviors among MSM population in different regions was different, with the highest in eastern China (26.5%) followed by central China (20.6%), northwest China (19.4%), northeast China (15.3%), south China (13.0%) and southwest of China (11.5%). MSM recruited through MSM venues and working groups showed a higher prevalence of heterosexual behaviors than VCT (37.9%, 25.9% VS 9.1%). The subgroup analysis on the sampling method showed the prevalence of heterosexual behaviors among MSM recruited by respondent-driven sampling (RDS). Snowball sampling and convenience sampling were similar, respectively, i.e. 23.1%, 22.5% and 22.5%. The prevalence of heterosexual behaviors among MSM of the self-administered questionnaires was greater than that of face-to-face interviews (27.5% vs. 17.4%). In the studies with the recall windows of 6 months, the prevalence of heterosexual behaviors among MSM was higher than that in those with the recall windows of 3 months (35.1% vs. 20.6%). The subgroups with larger sample size showed higher rates of heterosexual behaviors than that of the subgroups with small sample size. The estimated prevalence of heterosexual behaviors among MSM of studies before 2016 was greater than that after 2016.

**Table 2:** Subgroup analysis of heterosexual behavior in MSM population

Variables	Subgroups	The number of studies	The prevalence of heterogeneity behaviors among MSM and 95% CI	I^2^
Study locations	Northeast China	3	15.3% (8.30%, 27.5%)	98%
	Northwest China	1	19.4% (17.4%, 21.9%)	–
	East China	17	26.5% (23.1%, 31.0%)	96%
	Central China	4	20.6% (9.90%, 37.9%)	98%
	South China	11	13.0% (8.30%, 20.0%)	97%
	Southwest China	9	11.5% (7.40%, 17.4%)	97%
	Not reported	1	26.0% (22.5%, 29.6%)	–
setting	VCT	6	9.10 (6.5%, 12.3%)	79%
	MSM venues	5	37.9% (34.2%, 41.5%)	79%
	Social organization	3	13.0% (5.70%, 25.9%)	97%
	NGO	2	16.0% (13.8%, 18.0%)	0%
	Working group	2	25.9% (23.1%, 28.6%)	0%
	Multiple recruitment methods	18	15.3% (11.5%, 19.4%)	96%
	Not reported	10	25.4% (22.5%, 29.1%)	92%
Sampling method	Snowball sampling	18	22.5% (18.7%, 25.9%)	96%
	RDS	2	23.1% (12.3%, 38.7%)	94%
	Snowball sampling + RDS	4	17.4% (13.8%, 22.5%)	86%
	Convenience sampling	4	22.5% (13.0%, 36.3%)	97%
	Not reported or other	18	13.8% (9.9%, 19.4%)	97%
Survey method	Face-to-face	37	17.4% (15.3%, 20.0%)	97%
	Self-administered	21	27.5% (23.7%, 32.0%)	0%
	Web based	1	5.70% (3.80%, 8.30%)	–
	Not reported	6	27.5% (18.7%, 38.3%)	97%
Recall windows	3 momths	3	20.6% (18.0%, 23.7%)	79%
	6 months	42	35.1% (23.7%, 24.8%)	97%
	12 months	1	35.1% (29.6%, 40.5%)	-
Sample size	< 500	29	17.4% (13.8%, 21.3%)	96%
	500–1000	9	16.7% (11. 5%, 23.7%)	97%
	1000–1500	5	25.9% (18.7%, 34.6%)	98%
	> 1500	3	23.1% (18.7% , 28.6%)	95%
Study year	2016 ago	26	25.9% (25.4% , 26.5% )	97%
	Since 2016	11	16.7% (16.0% , 18.0%)	97%
	Not reported or others	9	25.4% (23.7 , 27.0%)	96%

### Sensitivity analysis and publication bias

**Fig. 3:**
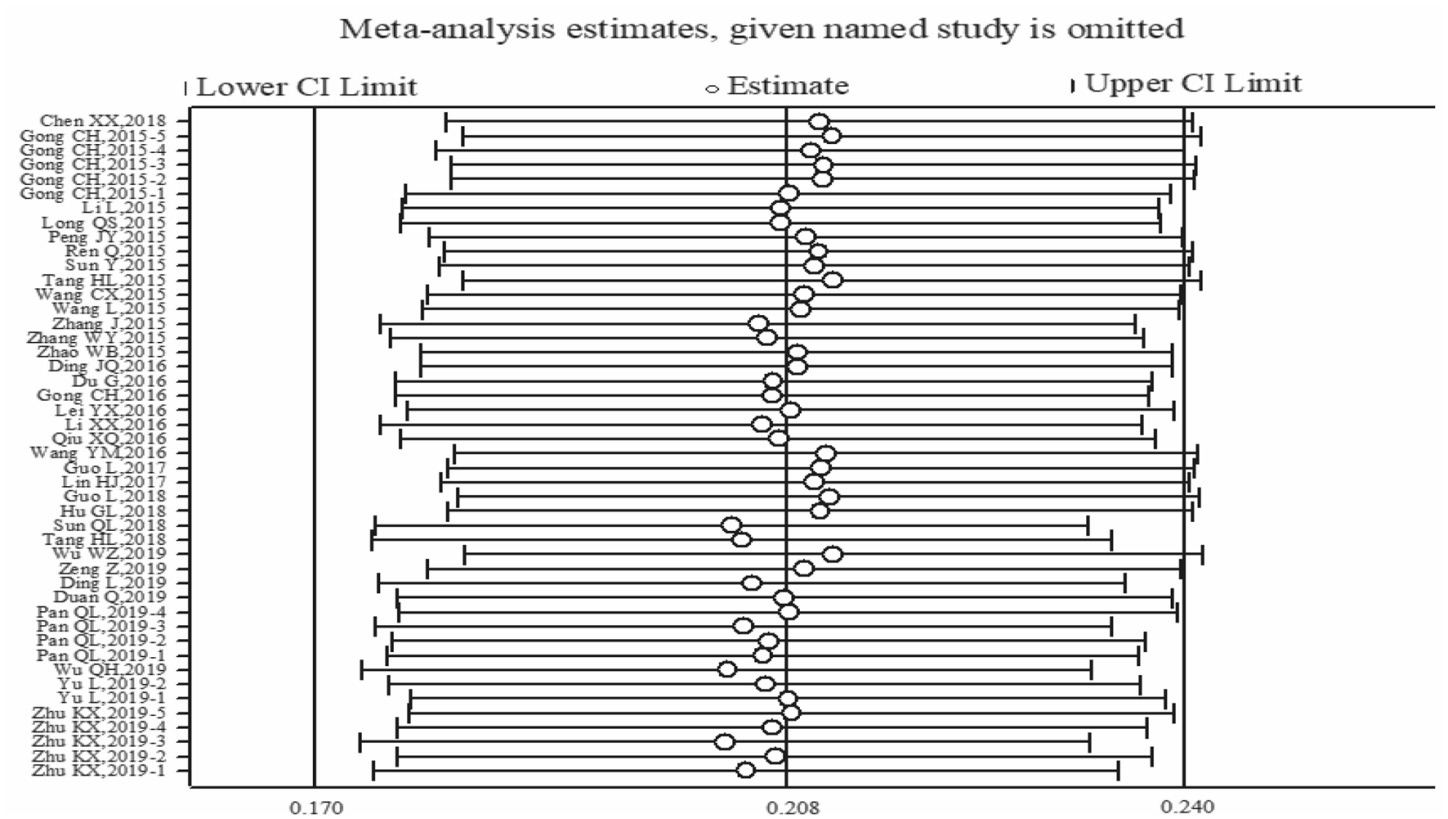
Sensitivity analysis diagram of the prevalence of heterosexual behaviors among MSM

**Fig. 4:**
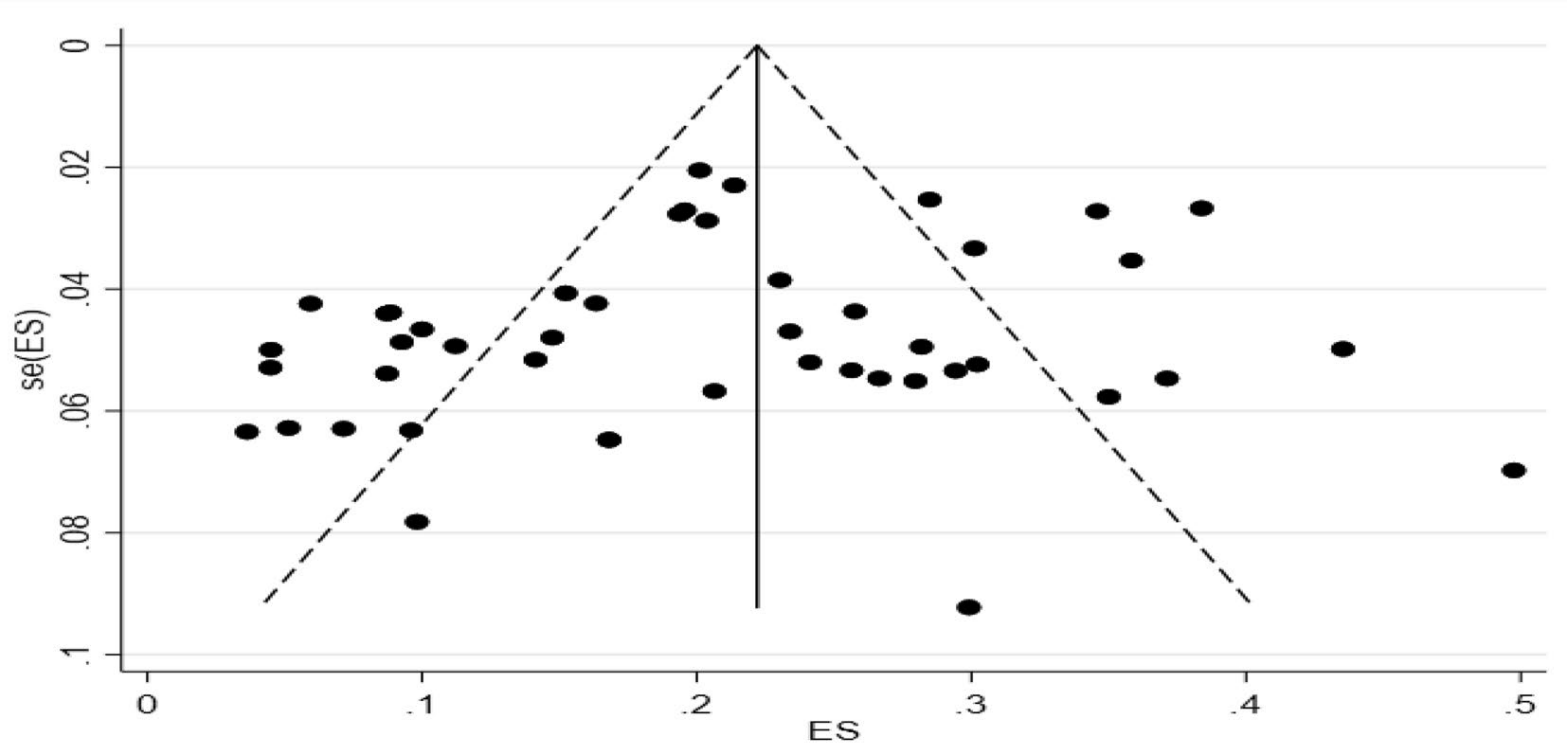
Funnel plot of ES, seES

It can be seen from the sensitivity analysis diagram that the included results were evenly distributed without significant deviation. The exclusion of a study at a time had no significant effect on the pooled prevalence, indicating that the aggregated results were robust and reliable. Figure 3 shows the sensitivity analysis diagram. The funnel plot in Fig. 4 is basically symmetrical, and further Egger’s test indicates that no publication bias was found in the study (*P* = 0.113 > 0.05).

### Condom utilization and HIV prevalence in MSM with heterosexual behaviors

**Fig. 5:**
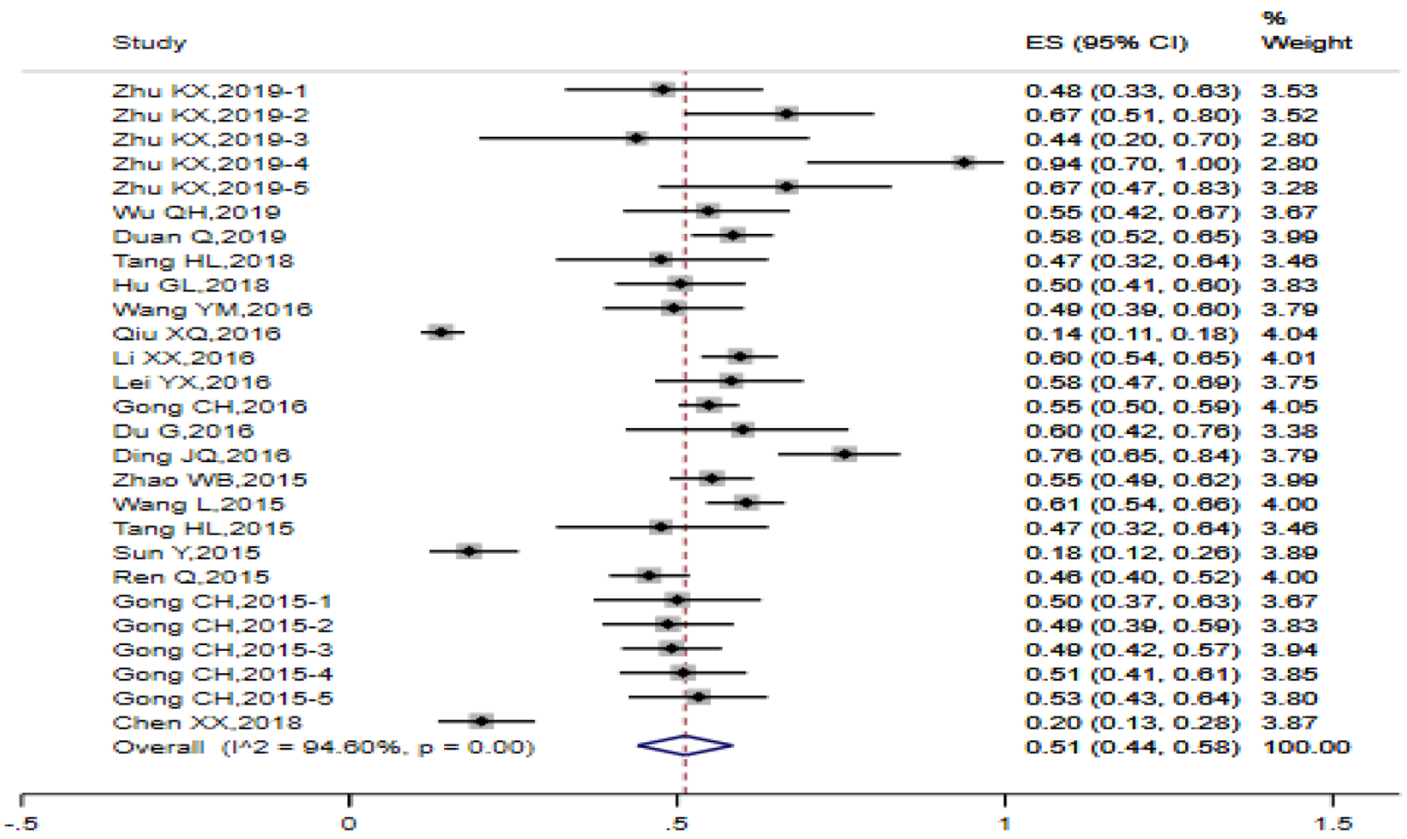
Forest chart of the condom utilization rate of the last heterosexual behavior among MSM

**Fig. 6:**
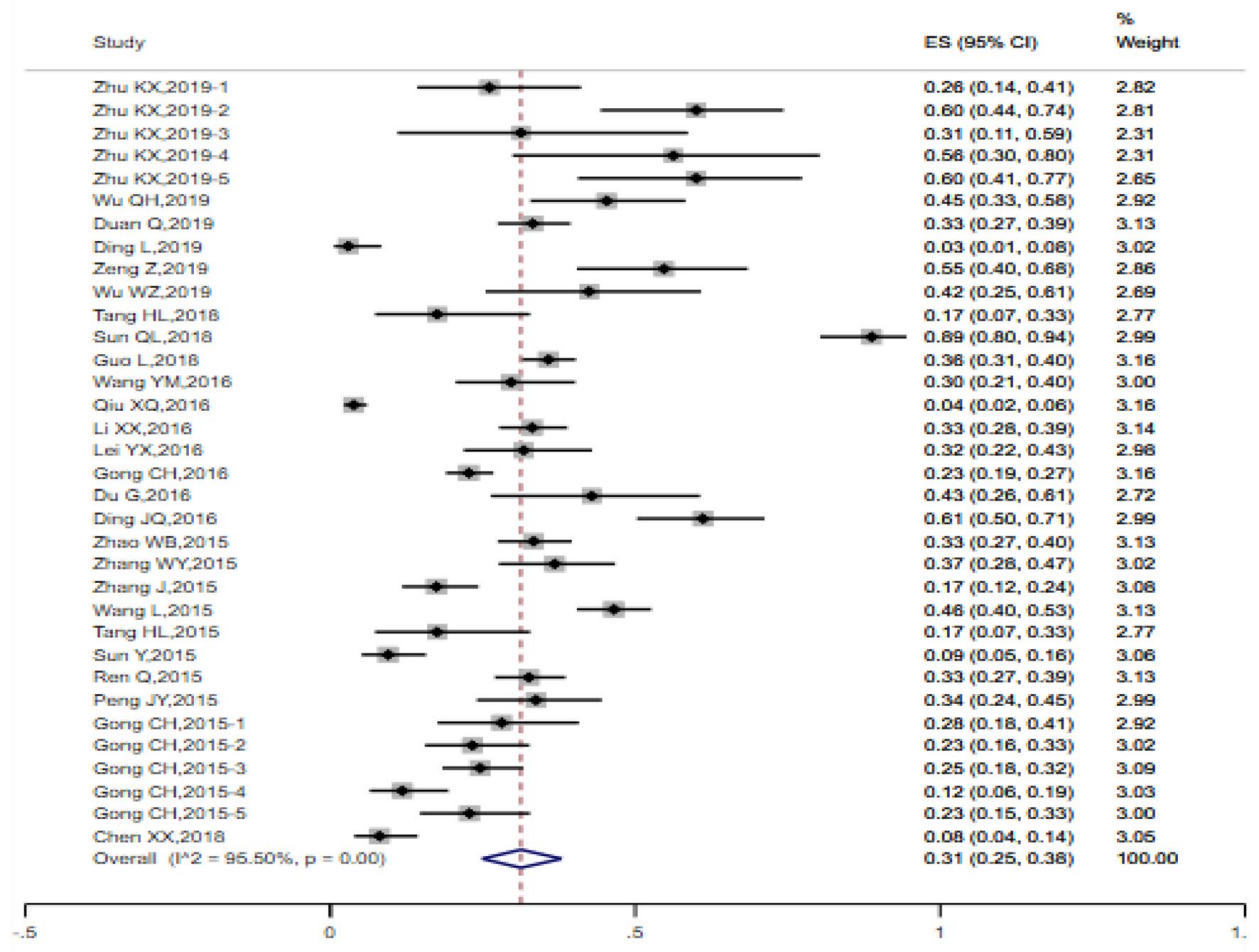
Forest chart of condom adherence rate of heterosexual behaviors among MSM

**Fig. 7:**
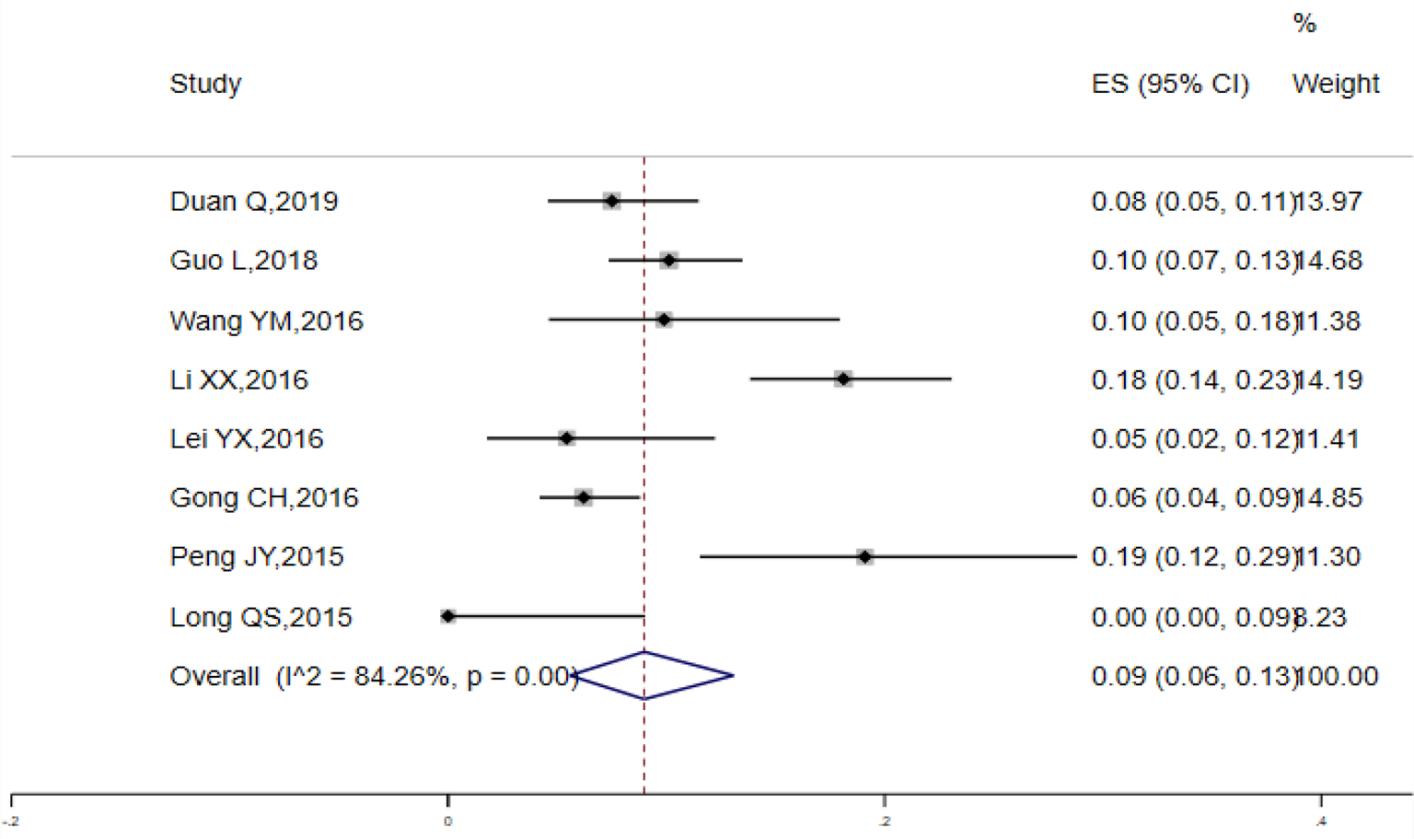
Forest chart of HIV pevalence in MSM with heterosexual behaviors

Random-effect model was employed to pool the condom utilization rate of the previous heterosexual behavior among MSM. The pooled rate among 27 studies was 0.51 (95% CI 0.44, 0.58) (I^2^ = 94.60%, Q test *P* < 0.0001) (Fig. 5). A total of 34 studies were incorporated into the analysis of condom adherence rate of heterosexual behaviors among MSM, with the estimated values 0.31 (95% CI 0.25, 0.38) (Fig. 6). The data obtained from 8 studies were included in the analysis of HIV prevalence in MSM with heterosexual behaviors and the estimated prevalence was 0.09 (95% CI 0.06, 0.13), There was a high degree of heterogeneity (I^2^ = 84.26%, Q test *P* < 0.0001) (Fig. 7).

## Discussion

To learn Chinese MSM population with heterosexual behaviors, we systematically reviewed the cross-sectional studies on this population, and the summarized prevalence of heterosexual behaviors among MSM was 0.19 (95% CI 0.17, 0.22), which was lower than the results of meta-analysis made by K Yun et al. [43]. They systematically reviewed the relevant studies before 2011, and concluded that the prevalence of heterosexual behaviors among MSM was 0.31 (95% CI 0.28, 0.35). Similarly, it was lower than the research results of Asia 0.328 [44]. The reason may lie in the social development and progress, as well as the influence of western countries’ attitudes towards homosexuality, all of these factors have resulted in a higher level of awareness and acceptance of MSM among Chinese people, which thereby led to the improvement of MSM people’s social recognition and less discrimination, and in turn the substantially decrease of the prevalence of heterosexual behaviors. However, the absolute number of MSM with heterosexual behaviors in China’s booming population was still large.

The pooled condom utilization rate of the last heterosexual behavior and condom adherence rate of heterosexual behaviors among MSM as well as their 95% CIs were 0.51 (0.44, 0.58) and 0.31 (0.25, 0.38), respectively, indicating that condom utilization rate was low in MSM population during heterosexual behaviors. These could be explained by the fact that MSM had insufficient awareness of safe sex, and the misunderstanding that AIDS and other sexually transmitted diseases were still far away from them. Besides, they believed that using condoms meant distrust. Many MSM’s regular heterosexual partners were wives, and the abandonment of condoms was taken as part of the measures to avoid the revealing of sexual orientation. In addition, heterosexual behaviors at uncertain time and place by MSM made condoms unavailable. Some MSM even thought that condom utilization should be considered by women.

The estimated HIV prevalence in MSM with heterosexual behaviors was 0.09 (95% CI 0.06, 0.13), which was higher than the meta analysis results obtained by K Yun et al. [43] in 2011 and Hongyi Wang et al. [45] in 2015. This may be due to the fact that HIV was more prevalent among MSM with heterosexual behaviors than before or that MSM’s greater access to HIV testing and treatment has led to an increase in detection and life. The HIV prevalence in MSM with heterosexual behaviors was also higher than that among men who have sex with men only [46, 47], suggesting that heterosexual behavior was a potential risk factor for HIV infection among MSM population.

In view of the extreme heterogeneity observed, we performed a subgroup analysis of the prevalence of heterosexual behaviors among MSM, and obtained some information from each subgroup. The prevalence of heterosexual behaviors among MSM varied from region to region. Specifically, the prevalence of heterosexual behaviors among MSM in eastern China was higher than that of other regions because it is a coastal region with developed economy, as well as dense and complex population with high mobility. Heterosexuality was more common among MSM recruited in the MSM venues, such as bars, clubs and baths, providing convenience for them to exchange information, and acting as the first choice for non-local MSM to find partners with high population mobility. This reminded us to pay great attention to these places, and carry out targeted AIDS prevention and treatment operation. On the contrary, the prevalence of heterosexual behaviors among MSM obtained by VCT was lower than that obtained by MSM venues, which is because that only those with definite awareness and attention to AIDS would come for the voluntary counseling test. Due to the privacy and confidentiality involved in face-to-face inquiry of the sexual behaviors of the target population, the report rate of heterosexual behaviors of the respondents was lower than that of the self-administered questionnaire.

Heterogeneity was not reduced according to the subgroup analysis, suggesting that there were additional sources of heterogeneity. MSM population was a relatively hidden population, and it was not easy to get enough samples through traditional probability sampling. Instead, non-probability sampling, such as snowball sampling, convenient sampling, time and locations sampling was widely adopted. It was doubtful whether the subjectively selected samples could represent the target population. Moreover, the MSM population was a very complicated group, that is, the age, education, marriage, occupation and income, as well as the mobility would all subtly affect and change the cognition and behaviors of the MSM population. However, the included studies came from 17 provinces across the country, and the above features of samples obtained were different, which may be the possible sources of heterogeneity. Nevertheless, we were unable to obtain the above detailed information from the included studies. It is expected to make use of probability sampling or conduct further accurate research on MSM population with a certain demographic characteristics in the future, and even explore MSM’s potential network in cooperation with multiple departments, which also pose a challenge to our work.

As we known, MSM groups characterized by anal or oral sex are high-risk groups for HIV infection. In recent years, it has been noted that male homosexual transmission was contributing to the rapid rise of AIDS in schools [48]. Migrant workers contribute to another high incidence of male sexual behaviors. “Money buys” makes male sexual behaviors more common, and the flood of inappropriate cartoons has a great influence on adolescents, most of them were driven by curiosity and motivated by peers to have homosexual behaviors [49]. Moreover, many MSM groups have heterosexual behaviors with at least one woman, and have minimal awareness of condom utilization. Therefore, AIDS may spread from MSM groups to female partners, and even further cause mother-to-child transmission.

In this regard, following suggestions were given: (1) Sufficient attention should be paid to AIDS protection by the MSM population, and it is reported that less than 50% of key population is covered by joint prevention services, indicating that these strategic groups are still marginalized and backward in terms of the anti-aids process [6]. Fortunately, China has included the MSM population as one of the priority groups for HIV/AIDS sentinel surveillance. The regular detection of HIV in MSM is in line with the concept of “the earlier detection,the earlier treatment”. If HIV positive people in MSM receive effective antiviral treatment until the HIV viral load continues to be undetectable, they will not pass HIV to women through sexual contact. We have to ensure adequate funding for the service to this population, and strengthen the scientific research of this population by carrying out more comprehensive and targeted work. (2) When it comes to MSM, people immediately and subconsciously associate it with AIDS, syphilis, gonorrhea and other diseases, making it “stigmatized”. This will cause behavioral and emotional problems in MSM [50], which is clearly wrong. What MSM needs objective understanding of them, respect to their freedom of sexual orientation, improvement of social acceptance, and the implementation of anti-discrimination laws. The societal prejudice against MSM people should be eliminated, especially the discrimination from medical staff when they seek medical treatment. All these measures will make the MSM population transparent and easy to manage, thereby improving the efficiency of AIDS publicity and investigation work. (3) The promotion of condom utilization is a low-cost and high-benefit intervention for MSM or the general population that needs to be improved, which is able to eliminate the concept that “condom utilization is distrust between each other” [51]. The policy of “Four Frees and One Care” policy needs to be more widely propagated and accepted. Moreover, attention should be paid to the appropriate ways of publicity and education, such as the establishment of partnership with the news media. Studies have shown that peer education has a significant impact on the behavior improvement of MSM population [52]. (4) The central role of community in AIDS prevention and treatment of the MSM population should be strengthened, thereby making full use of the positive guiding role of community in MSM behaviors [53].

## Limitations

This meta-analysis suffered certain limitations. At first, we did not search more studies by back-tracking references, which might leave out eligible studies. Secondly, the included studies were cross-sectional studies ones, and the demographic characteristics of the targeted population had a significant impact on the effect indicators, therefore, the inter-study heterogeneity was high. Moreover, the cross-sectional study failed to consider the sequence of time, which could not determine the causal relationship. In that case the risk of HIV prevalence in MSM with heterosexual behaviors has to be confirmed by abundant and high-quality cohort studies in the future. Thirdly, due to the limited availability of health resources, the heterosexual behaviors survey of MSM population in rural areas had not been paid enough attention and carried out. Therefore, the included studies were only limited to the urban areas, and the indicators obtained by the research could not represent the national situation.

## Conclusions

To sum up, Chinese MSM population with heterosexual behaviors accounts for a large proportion. Their low condom utilization, soaring HIV infection and special sexual behaviors make them a high-risk group for HIV transmission. We should pay adequate attention to this special group and take measures to reduce their risk of HIV transmission from sub-population to the general population.

## Data Availability

All data generated or analysed during this study are included in this published article.
